# IFITM3 promotes bone metastasis of prostate cancer cells by mediating activation of the TGF-β signaling pathway

**DOI:** 10.1038/s41419-019-1750-7

**Published:** 2019-07-04

**Authors:** Xi Liu, Lu Chen, Yinghui Fan, Yi Hong, Xiaoqun Yang, Yao Li, Jianlei Lu, Jianmin Lv, Xiuwu Pan, Fajun Qu, Xingang Cui, Yi Gao, Danfeng Xu

**Affiliations:** 1 Department of Urology, Ruijin Hospital, School of Medicine, Shanghai Jiaotong University, Shanghai, China; 2 Department of Anesthesiology, Renji Hospital, School of Medicine, Shanghai Jiaotong University, Shanghai, China; 3 Department of Urology, Changzheng Hospital, Second Military Medical University, Shanghai, China; 4 Department of Urology, Third Affiliated Hospital, Second Military Medical University, Shanghai, China

**Keywords:** Prostate cancer, Cancer metabolism

## Abstract

Advanced-stage prostate cancer (PCa) is often diagnosed with bone metastasis, for which there are limited therapies. Transforming growth factor β (TGF-β) is known to induce epithelial–mesenchymal transition (EMT), and abundance of TGF-β in the bone matrix is one of the important growth factors contributing to bone metastasis. TGF-β is reported as a key mediator of bone metastasis, but the underlying mechanism has not been elucidated. It was found in our study that Interferon-inducible Transmembrane Protein 3 (IFITM3) played a key role in the regulation of malignant tumor cell proliferation, invasion, and bone migration by binding to Smad4, thus activating the TGF-β-Smads Signaling Pathway. Lentivirus-mediated short hairpin RNA (shRNA) knockdown of IFITM3 inhibited cell proliferation and colony formation, induced apoptosis and inhibited migration by reversing EMT and downregulating the expression of metastasis-related molecules including FGFs and PTHrP. Microarray analysis showed that IFITM3 knockdown could alter the MAPK pathway associated with TGF-β-Smads signaling. By knocking down and overexpressing IFITM3, we demonstrated that IFITM3 expression level had an effect on MAPK pathway activation, and this change was more pronounced upon exogenous TGF-β stimulation. These results suggest that IFITM3 played an oncogenic role in PCa progression and bone metastasis via a novel TGF-β-Smads-MAPK pathway.

## Introduction

Prostate cancer (PCa) remains one of the most universal malignant tumors and the major cause of cancer-associated death in the male worldwide^1^. About 90% cancer-related deaths were attributed to metastasis. The bone is the most common site of PCa metastasis^2^. About 70% of advanced PCa patients were diagnosed with bone metastases. PCa, with breast cancer (BCa) together, takes up over 80% cases of bone metastatic disease^3^. Early screening makes it possible for most patients to detect the PCa in local stage, and a considerably good prognosis and long-term survival can be achieved by regular treatment. Whereas, once the patients have PCa biochemical recurrence, most of them may eventually develop into metastatic PCa. This subset of patients are more likely to transform into castration resistant prostate cancer, though the rational use of androgen deprivation therapy may retard the progression of disease in locally advanced or metastatic PCa patients to some extent, but the prognosis is still usually poor because of limited treatments for such patients. Therefore, identification of effective biomarkers for understanding the molecular mechanisms of bone metastatic signaling pathways underlying PCa would help improve the therapeutic outcome and prognosis.

Transforming growth factor β (TGF-β) acts as a suppressor in precancerous cells, but as a metastatic promoter in cancer cells. Due to the block effect of the cell cycle, TGF-β has tumor suppressor function in normal and precancerous tissues. However, large quantities of TGF-β secreted from tumor cells and the local microenvironment promote the invasion and metastasis of various tumors through an autocrine and paracrine mechanism^4^. TGF-β induces epithelial–mesenchymal transition (EMT) in which epithelial tumor cells acquire aggressive mesenchymal-like phenotypes accompanied with alterations in the expression of intercellular adhesion molecules and metalloproteinase secretion, resulting to metastasis^5^. Of note, TGF-β is also the key mediator of bone metastasis. The intricate two-way interaction between cancer cells and the bone microenvironment promotes osteolytic lesio and occurrence of bone metastasis^6^.

Interferon-inducible Transmembrane Protein 3 (IFITM3) is a member of the IFITM gene family that acts as a key role in immune cell signaling, cell adhesion, and stem cell migration^7,8^, which is involved in regulating primal germ cell migration, hepatitis C virus infection, and endodermal localization, as well as mediating the regulation of endothelial cells, adhesion, and apoptosis of leukocytes and other biological processes^9–11^. In addition to these biological effects, the role of the IFITM3 gene in tumors is increasingly emphasized. It was found that IFITM3 gene expression was abnormally elevated in colon cancer tissue compared to normal colon tissue, and significantly higher in metastatic lymph nodes than that in cancer and normal colon tissues^12^. Downregulate IFITM3 expression level could significantly restrain the proliferation, migration, and invasion in colon cancer cells and also inhibit tumor growth and metastasis in animal models. IFITM3 is not only an invasive gene in colon cancer but highly expressed in most cancers such as breast cancer, glioma, melanoma, gastric cancer, and cervical cancer. It is positively correlated with the degree of tumor pathological differentiation and is involved in tumorigenesis, progression, relapse, and the transfer process^13–16^. Nevertheless, the functional roles of IFITM3 in PCa remain poorly understood. The purpose of this study was to investigate the functional significance of IFITM3 and the novel carcinogenic pathways that this molecule regulates in PCa cells.

Here, we focused on the TGF-β-mediated mechanism of PCa bone metastasis, hoping to find the site of regulating the TGF-β signaling pathway activity. The result showed that IFITM3 knockdown significantly inhibited tumor cell migration and invasion, and this inhibitory effect was more pronounced in TGF-β pathway activated cells. In addition, IFITM3 could affect TGF-β signaling activation by binding to Smad4 protein. Discovery of the molecular targets and pathways regulated by oncogene IFITM3 will explore new potential molecular mechanisms of PCa carcinogenesis and metastasis, thus facilitating the development of novel therapeutic strategies for bone metastasis in PCa patients.

## Materials and methods

### Clinical specimens and cell culture

The tissues needed in the experiment were taken from the specimens after radical prostatectomy in the urology department of Changzheng hospital (Shanghai, China). The samples were taken according to the following standard steps: 1. Observe the prostate specimens and distinguish the anatomical parts of the specimens. 2. Prostate was cut along the coronal plane and divided into 6–8 pieces. The thickness of the single piece was about 5 mm. 3. Observe with naked eye, select hard texture, unclear boundary, white or gray cut surface, small lesions with small yellow spots to be taken as PCa tissue specimens, adjacent normal prostate tissue to be taken as matched paracancerous tissue specimens, and pay attention not to damage the integrity of the capsule of the general specimens. 4. Tissue samples were labeled, half of them were embedded in paraffin to make slices, and the other half were preserved in liquid nitrogen. The diagnosis of PCa tissue or adjacent normal prostate tissue was finally confirmed by pathologist. Six pairs of PCa and adjacent tissues cryopreserved in liquid nitrogen were randomly selected for qRT-PCR detection within 3 months. Additional slices of 50 PCa tissues, 37 adjacent tissues, and 14 bone metastatic tissues (obtained by needle biopsy) were collected for immunohistochemistry. All patients provided informed consent with regard to tissues collected, and the experimental design was approved by the Ethics Committee of the Second Military Medical University (Shanghai, China). DU145 cells were cultured in Ham’s F-12 nutrient mixture (Gibico) with 1 mM nonessential amino acids, 10% fetal bovine serum, 100 U/mL penicillin, and 100 μg/mL streptomycin. PC-3 was maintained in the same manner as DU145 but did not include nonessential amino acids. Cells were humid cultured in a 37 ℃ 5% CO_2_ incubator. PCa cell lines were obtained from the Cell Bank of the Chinese Academy of Sciences.

### Immunohistochemistry

The paraffin blocks were sliced into 4-μm-thick sections, and the process of section dewaxing was performed in xylene and different concentrations of ethanol, incubated with 1:100 anti-IFITM3 at 4 ℃ overnight. Then the sections were incubated with generally biotinylated goat anti-rabbit serum and streptavidin–peroxidase conjugate for 15 min at room temperature and finally stained with diaminobenzidine.

### Reagents and antibodies

3-(4,5-dimethyl-2-thiazolyl)-2,5-diphenyl-2-H-tetrazolium bromide (MTT), dimethyl sulfoxide (DMSO), and SB431542 were from Sigma Chemical Co. (St. Louis, MO, USA); TGF-β was provided by Novoprotein Scientific Inc. (Shanghai, China); IFITM3 primary monoclonal antibody were from Sigma Chemical Co.; P38-MAPK and p-P38, Smad and p-Smad, CDC2 and P-CDC2, mTOR and p-mTOR primary monoclonal antibodies were purchased from Cell Signal Technology, Inc (Beverly, MA, USA); E- and N-cadherin primary monoclonal antibodies were from Abcam Ltd. (Cambridgem MA, USA).

### Lentivirus construction and transfection

Small interfering RNA (siRNA) targeting IFITM3 (NM_021034) and nonsense control siRNA sequences were transformed into stem-loop-stem oligonucleotides (shRNA). The targeted and messy siRNA sequences are 5′-CCTCATGACCATTCTGCTCAT-3′ and 5′-TTCTCCGAACGTGTCACGT-3′, respectively. ShRNA was inserted into the pGreenPuro vector, and DNA sequencing was used to confirm the vector expression. The shRNA expression vector (pVSVG-1) and packaging vector (pCMVMR8.92) were cotransfected to 293 T cells using Lipofectamine 2000. After 48 h, the supernatant was filtered through a 0.45 μm cellulose acetate filter and finally centrifuged for 1.5 h in a 50,000 *g* ultracentrifuge to concentrate the virus. A total of 5 × 10^4^ cells/well of DU145 and PC-3 cells were infected with lentivirus for ∼72 h expressing IFITM3 shRNA (Lv-shIFITM3) or control shRNA (Lv-shCon). The multiplicity of infection was 30 and 35, respectively.

### Expression vectors and reporter constructs

The vectors expressing GFP (pCDH-CMV-MCS-EF1-copGFP) and (pCDH-CMV-MCS-EF1-puro) were obtained from Cambridge Bioscience. Primers F (5′-gaattcATGAATCACACTGTCCAA-3′) and R (5′-cccgggCTACCCGGGTCCATAGGCCTG-3′) were designed to amplify IFITM3 gene(NM_021034), and primers F (5′-gaattcATGGACAATATGTCTATT-3′) and R (5′-cccgggGACCCACAACCTTTAGAC-3′) were designed to amplify Smad4 gene(NM_005359.5). Primer F and primer R contain Xba I and Xma I restriction sites, respectively. To generate pCDH-CMV-Flag-MCS-IFITM3-EF1-copGFP, pCDH-CMV-Flag-MCS-IFITM3-HA-EF1-copGFP, pCDH-CMV-Flag-MCS-Smad4-EF1-copGFP, and pCDH-CMV-Flag-MCS-Smad4-HA-EF1-copGFP, the corresponding cDNA sequences were inserted in pCDH-CMV-MCS-EF1-copGFP. To avoid the interference of green fluorescence in the immunofluorescence (IF) experiments, we also constructed the plasmid with the above cDNA sequences and inserted it in pCDH-CMV-MCS-EF1-puro vector. The target PCR fragment recovered with the Agarose Gel DNA Extraction Kit GC-3U (Gene Ray) was ligated to the corresponding vector and transformed into E. coli DH5a. The positive clones were selected to Gene Ray for sequencing. #1 and #2 mean the different IFITM3 overexpressing monoclonal cell lines selected. The mainly used primer sequences: Flag F primer: ctaggATGGATTACAAGGATGACGACGATAAGt; R primer: ctagaCTTATCGTCGTCATCCTTGTAATCCATt; HA F primer: tcgagTACCCATACGATGTTCCAGATTACGCTTAGgc; R primer: ggccgcCTAAGCGTAATCTGGAACATCGTATGGGTAc.

### Cell proliferation and colony formation assay

The treated cells were trypsinized, suspended, and seeded in 96-well plates (with five replicate wells) at a density of 2000 cells/well. On day 1–5, 20 μL MTT solution (5 mg/mL) was added, and after 3 h, using 100 μL acidified isopropanol to terminate the reaction. The absorbance at 570 nm of each well was measured using a spectrophotometer. For cell colony formation, the same-treated cells were seeded at a density of 200 cells/well in 96-well plates (three wells per group). The cells in plates were cultured for 7–14 days with the medium changed every 3 days. When observing the results, the cells were fixed in 4% paraformaldehyde for 30 min and stained with GIEMSA dye (Sigma) for 20 min.

### Cell cycle and apoptosis analysis

For cell cycle analysis, trypsin-digested cells were washed with ice-cold PBS, fixed with 950 μL ice-cold 75% ethanol for 1 h, and then incubated with freshly prepared PI staining solution at 37 ℃ for 1 h in the dark. For apoptosis analysis, cells were resuspended and double stained with annexin-V/7-ADD for 30 min at 4 ℃ in the dark. Each experiment was repeated three times. Cell cycle distribution and apoptosis were analyzed by flow cytometry using specific software (FACS Calibur, BD, San Jose, CA).

### Transwell migration assay

Cells successfully infected with the lentivirus were trypsinized, washed, and resuspended in 0.1% BSA. Then evenly inoculated into the upper compartment, and incubated for 24 h in a cell culture incubator. Subsequently, the lower compartment cells were fixed with a 10% methanol solution for 0.5 min after removing the upper compartment cells and then stained with crystal violet for 20 min. After washing with ddH_2_O, cells were observed under an optical microscope. Each experiment was repeated three times.

### Western blot and quantitative real-time PCR

The cells were fully lysed with 0.5 mL ice-cold lysis buffer (pH 7.4, 50 mM Tris-HCl, 4% SDS, 20% glycerol, and 2% mercaptoethanol). Then collecting the supernatant and using the BCA assay to determine the protein concentration. The lysed proteins were separated on an SDS-PAGE gel and transferred to a PVDF membrane for immunoblotting analysis. The membrane was immersed in a 40–50 mL TBST solution containing 5% nonfat milk at room temperature for half an hour and incubated with the primary antibody. After TBST washed three times, the transferred membranes were incubated with 1:5000 horseradish peroxidase-conjugated goat anti-rabbit IgG antibody at room temperature for 1 h. Proteins were detected in ECL plus^TM^ System. For qPCR reactions, the relative expression level was calculated using the 2^–ΔΔCt^ method on the basis of β-actin for normalization and the primer sequences used are listed in Table 1. Each experiment was repeated three times.

**Table 1:** The primers used for qRT-PCR reactions

	Forward (5′–3′)	Reverse (5′–3′)
IFITM3	TAGGGACAGGAAGATGGTTGG	GGATGACGATGAGCAGAATGG
FGF1	GATGGCACAGTGGATGGGAC	GATGGCACAGTGGATGGGAC
FGF2	GCCTTCTCTTTCAGCATTCACACC	AGCCAACTCGTAACAATCCATCAG
Fibronectin	AATTGCTAGTTTACCGTTCAGAAG	ATGAAGGAAAGGTGGAGGGAAG
MMP2	AAAATGGATCCTGGCTTCCC	AATAGGCGCCCTTGAAGAAGT
MMP9	TTGGTCCACCTGGTTCAACT	ACGACGTCTTCCAGTACCGA
α-SMA	CTGTTCCAGCCATCCTTCAT	TCATGATGCTGTTGTAGGTGGT
Collagen1A2	ATGAGGAGACTGGCAACCTG	GGCGTGATGGCTTATTTGTT
PAI-1	AAACTCCCTAGTCTCCACCTGA	CCTTAAGGGAGTTGTGCTTCA
Snail	CCAGTGCCTCGACCACTATG	CTGCTGGAAGGTAAACTCTGG
CTGF	CTCCTGCAGGCTAGAGAAGC	GATGCACTTTTTGCCCTTCTT
PTHrP	GTCTCAGCCGCCGCCTCAA	GGAAGAATCGTCGCCGTAAA
β-actin	ATCGTGCGTGACATTAAGGAG	AGGAAGGAAGGCTGGAAGAG

### Coimmunoprecipitation assays and IF

To clarify the proteins associated with IFITM3, HEK293T cells transfected with pCDH-CMV-Flag-MCS-EF1-puro plasmid encoding IFITM3 and pCDH-CMV-HA-MCS-EF1-puro plasmid encoding Smad4 were lysed in lysis buffer (20 mM MOPS pH 7.5, 0.15 M NaCl, 0.5% CHAPS, 1 mM EDTA, 1 mM dithiothreitol, and protease inhibitor cocktail). Monoclonal ANTI-FLAG^®^ M2 (F1804,sigma), anti -HA (11666606001,Roche), and Magne Protein G Beads (G7471,Promega Corporation) were used for FLAG and HA pulldown experiments. Freshly isolated HEK-293T cells expressing Smad4-HA and Flag-IFITM3 were subjected to IF staining. Cells were fixed 30 min with 4% paraformaldehyde at 37 °C. The smear was permeabilized with 0.5% Tween 20 (Bio-Rad Inc.) and blocked with 10% normal goat serum and then incubated with the corresponding primary antibody overnight at 4 °C. Subsequently, the cells were incubated with the corresponding secondary antibody in the dark: anti-Mouse IgG−FITC antibody (F9137,Sigma), anti-Rat IgG (H + L) cross-adsorbed secondary antibody, and PE(A10545,thermo fisher). After termination, the sections were counterstained with DAPI solution, mounted, and observed using an inverted fluorescence microscope (Olympus, Japan).

### Microarray screening

Using Trizol to extract RNA from the PC-3 cells which transfected with the IFITM3 plasmid (Lv-shIFITM3) and the negative control plasmid, respectively. Total RNA content was quantified using the BioTek Epoch system. Microarray analysis was completed and technically supported by OE Biotech Co., Ltd. (Shanghai, China). The differential genes were screened after probe filtration and at least one set of 100% labeled detection probes were selected in each set of samples for subsequent analysis. For analyses without biological replicates, only the difference fold change values were used for screening, and the threshold was fold-change value ≥2.0. GO analysis of differential genes to describe the function of this gene. Pathway analysis of differential genes was performed using the KEGG database, and statistical significance was used to calculate the significance of differential gene enrichment in each pathway.

### Animal experiments and bioluminescence imaging

Four male nude mice were randomly divided into two groups and injected with 5 × 10^6^ PC-3 cells via the tail vein. The cells were infected with Lv-shCon or Lv-shIFITM3 lentiviruses which reconstruct of a luciferase reporter. One male nude mice was injected with normal PC-3 cells as a control group without fluorescence. The in vivo inhibitory effect of silencing IFITM3 on cell migration was evaluated by measuring the luciferase intensity of human PC-3 xenografts grown in 4 weeks after tail vein injection. For bioluminescence imaging in vivo, 30 min before imaging, animals were given an IP injection of 150 mg/kg D-luciferin in PBS. Quantitation of fluorescence and bioluminescence signals was using Living Image 4.2 software (Caliper Life Sciences, Hopkinton, MA, USA).

### Statistical analysis

Data are expressed as the mean ± SD. The results were subjected to the Student’s *t*-test using SPSS software (20.0 version), *P*-value < 0.05 was considered statistically significant (*P < 0.05, **P < 0.01; ***P < 0.001).

## Results

### IFITM3 is overexpressed in PCa tissues and correlated with Gleason score and T stage

**Fig. 1:**
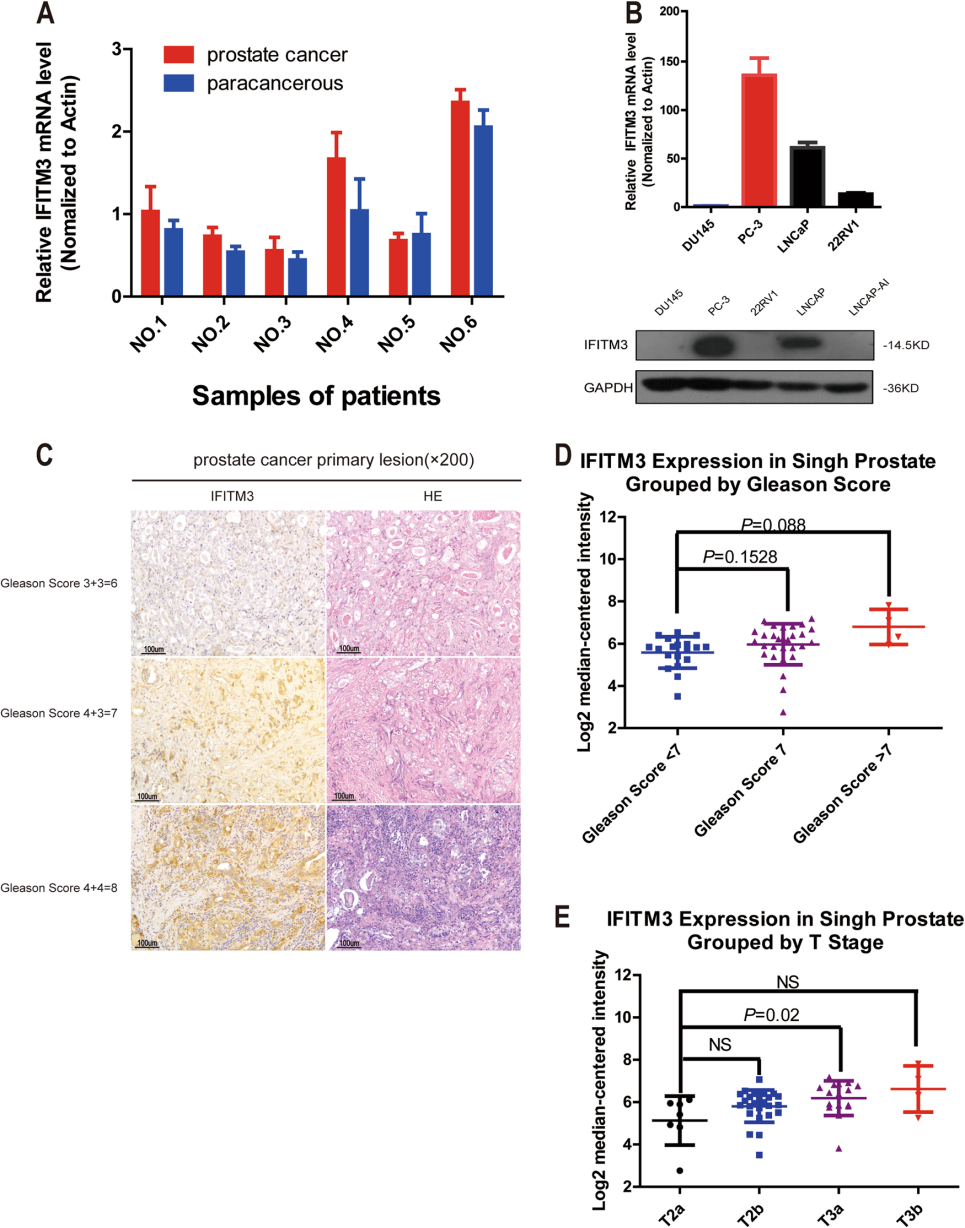
IFITM3 overexpression in PCa tissues is correlated with Gleason score and T-stage as shown by qRT-PCR, IHC, and database analysis. **a** IFITM3 mRNA expression in PCa vs. paracancerous tissues by qPCR. **b** The expression level of IFITM3 in PCa cell lines by qPCR and Western blotting. **c** Representative immunohistochemical images showing IFITM3 expression in PCa tissues of different Gleason scores. **d**, **e** According to Singh Prostate of Oncomine database, a high mRNA expression of IFITM3 suggests a higher Gleason score and is associated with an advanced T-stage

The expression level of IFITM3 in the six pairs of PCa and adjacent tissues was analyzed by qRT-PCR method. The result demonstrated that the expression level of IFITM3 was significantly higher in PCa tissues than that in adjacent tissues (Fig. 1a). Western blotting revealed that IFITM3 protein was commonly expressed in PCa cell lines (Fig. 1b). The cancer and adjacent tissues of PCa patients were collected for immunohistochemical analysis to detect the expression level of IFITM3. As shown in Table 2, double-blind scoring of 87 tissue sections by pathologists confirmed the increased expression of IFITM3 in PCa, and the difference was considered to be statistically significant (*P* < 0.001). Meanwhile, with the PCa tissue Gleason score increasing, IFITM3 gradually deepened and more extensively stained (Fig. 1c). The representative database also shows that there is a high level of IFITM3 expression in the advanced PCa organization (values are presented in log2 median-center intensity information). Singh Prostate of Oncomine (a cancer microarrays database) (Fig. 1d) suggests that IFITM3 expression was positively correlated with Gleason score in PCa tissues (Gleason score < 7 vs. =7: *P* = 0.1528; Gleason score < 7 vs. >7: *P* = 0.088). In TNM Classification of Malignant Tumors, we also found that the expression of IFITM3 was elevated with T-staging progress (T3a vs. T2a: *P* = 0.02) (Fig. 1e).

**Table 2:** IHC staining of IFITM3 expression in PCa tissues

	−	+	++	Case	The rate of positive	*P* ^a^	*P* ^*b*^
Paracarcinoma	30 (81.1%)	7 (18.9%)	0 (0%)	37	18.9%	<0.001	<0.001
Carcinoma	20 (40.0%)	21 (42.0%)	9 (18.0%)	50	60.0%		

^a^Mann–Whitney *U* test

^b^Wilcoxon signed-rank test

### Knockdown efficiency of IFITM3 gene

**Fig. 2:**
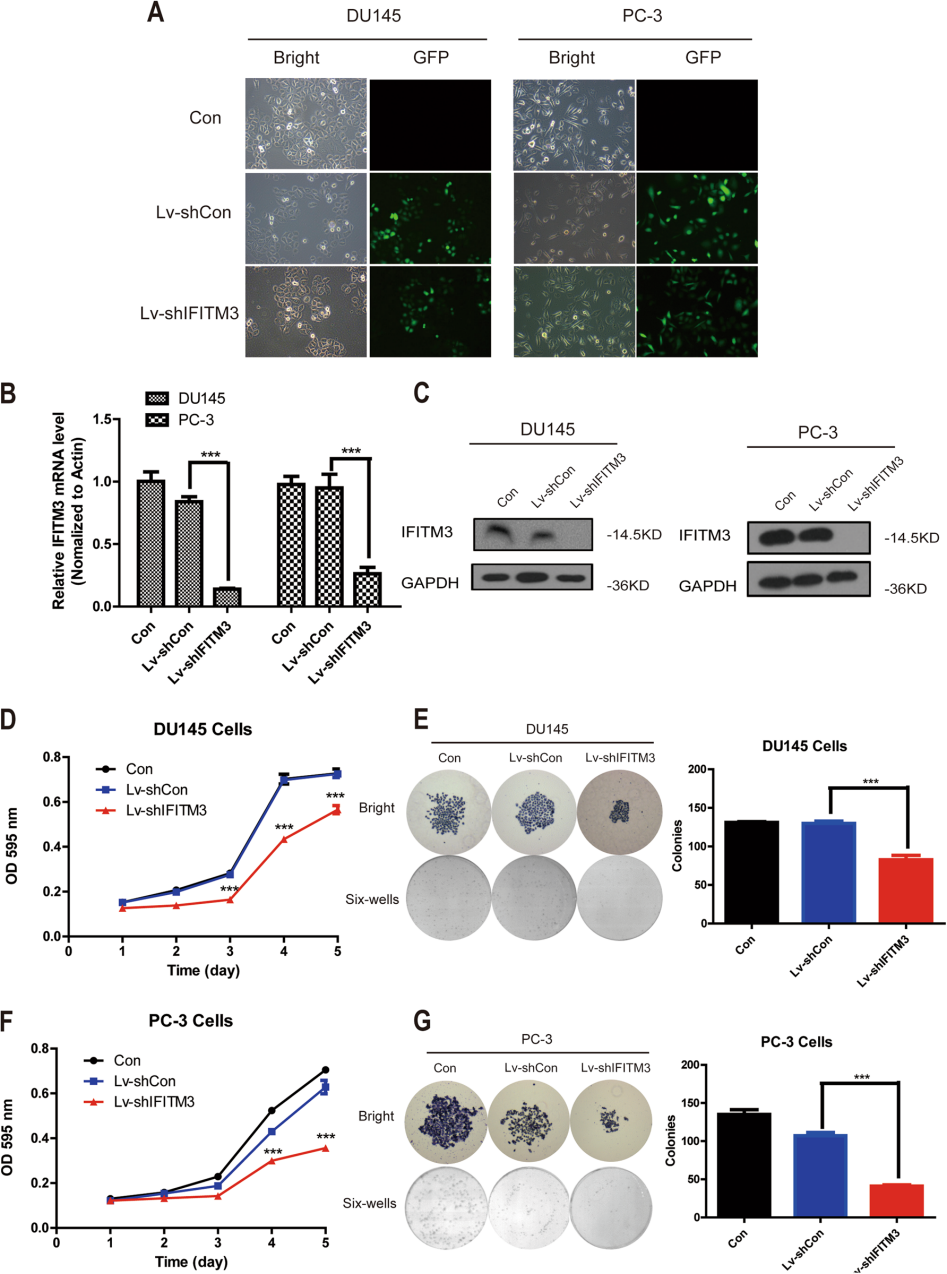
Silencing IFITM3 inhibited colony formation and proliferation in DU145 and PC-3 cells. **a** Lentivirus-mediated knockdown of IFITM3 in DU145 and PC-3. The lentivirus was successfully transfected and the result was measured in bright and GFP (magnification × 100). **b**, **c** The silencing efficiency of IFITM3 was assessed by qPCR and Western blot in DU145 and PC-3 cells. **d**, **f** The proliferation of PC-3 and DU145 was obviously inhibited after IFITM3 silencing by MTT method. **e**, **g** The size and number of colonies for DU145 and PC-3 cells were significantly smaller in shIFITM3 group as compared with shCON group

DU145 and PC-3 cells were chosen to be infected with the recombinant lentiviral vector containing siRNA against IFITM3 or control siRNA and GFP expression showed successful lentiviral infection after 4 days (Fig. 2a). According to the result of qRT-PCR, the knockdown efficiency of IFITM3 gene in DU145 and PC-3 cells was 83.4% and 72.3%, respectively (Fig. 2b), meaning that the expression of IFITM3 related mRNA was significantly lower in Lv-shIFITM3 group than that in the other control groups. Meanwhile, the knockdown efficiency of IFITM3 was detected by Western blotting. The results suggested that the expression level of IFITM3 in DU145 and PC-3 cells of Lv-shIFITM3 group was lower than that of Lv-shCON group (Fig. 2c), demonstrating that IFITM3 was knocked down efficiently and could be used in subsequent experiments.

### IFITM3 silencing inhibits proliferation and colony formation by inducing apoptosis in PCa cells

**Fig. 3:**
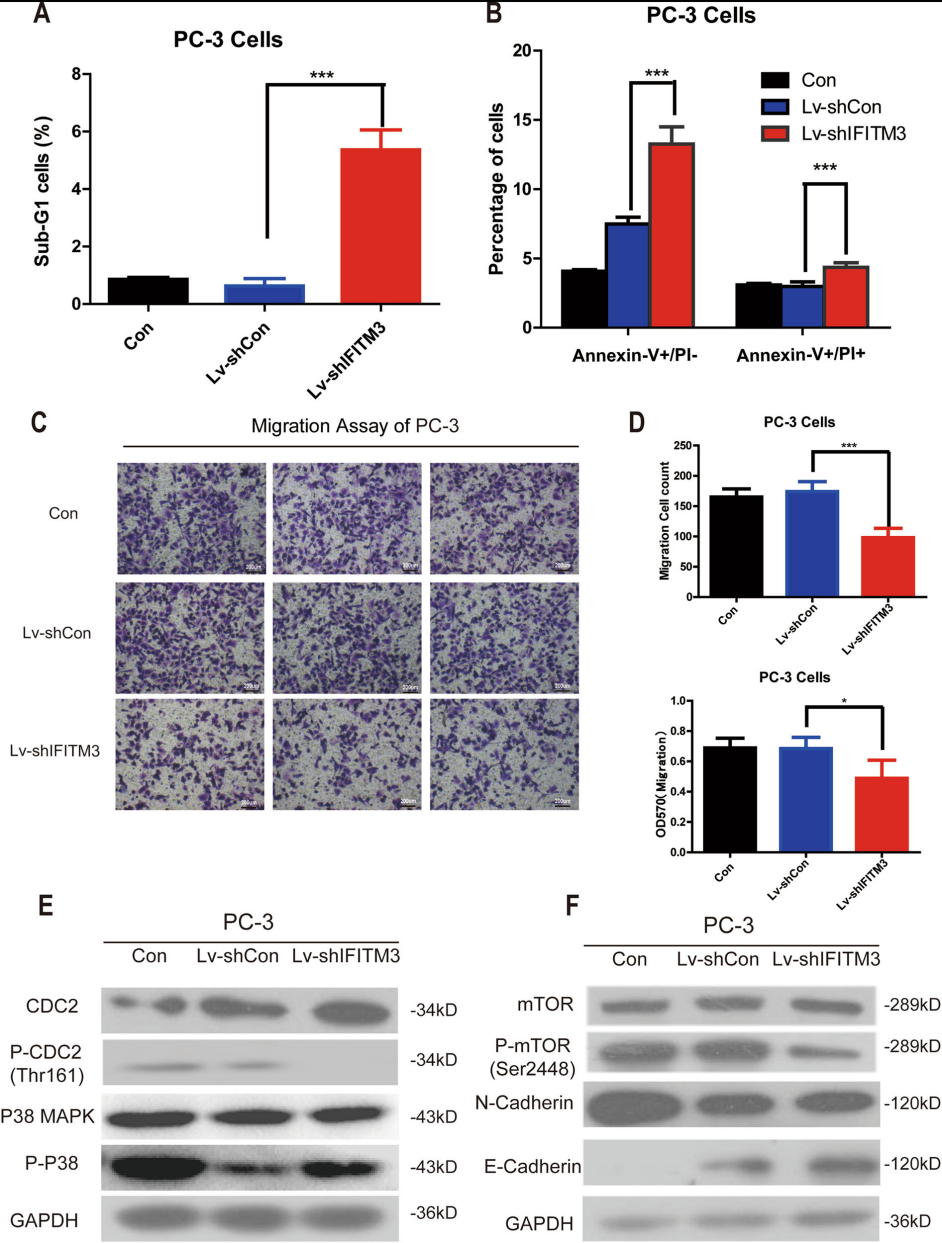
IFITM3 knockdown induces apoptosis and inhibits cell migration via MAPK and EMT. **a** IFITM3 silencing induced apoptosis as represented by Sub-G1 accumulation in PC-3 cells. **b** The number of early apoptosis (V+/PI−) and late apoptosis (V+/PI+) cells stained by Annexin-V in Lv-shIFITM3 group was significantly greater than that in the control group. **c** The number of cells migrating through the 8 μm diameter pores in Lv-shIFITM3 group was decreased significantly compared with the control group. **d** PC-3 cell counts and the absorbance at 570 nm of Lv-shIFITM3 group were significantly lower than those in the control group. **e** Western blot results of CDC2/P-CDC2 and P38/P-P38 in PC-3 cells with or without IFITM3 silencing. **f** Western blot analysis showing decreased phosphorylation of mTOR and N-cadherin, and increased E-cadherin in PC−3 cells after infection with Lv-shIFITM3 vs. Lv-shCon. GAPDH was used as loading control. Western blot data presented were representative of those obtained in at least three separate experiments

MTT assay showed that the proliferative activity of DU145 and PC-3 cells was decreased after sh-IFITM3 transduction (***P < 0.001) (Fig. 2d, f). At the same time, experiments by plate colony formation also showed that knocking down IFITM3 in DU145 and PC-3 cells resulted in a significant decrease in the size and number of colonies (Fig. 2e, g). Further, FACS was performed to assess cell cycle distribution and to speculate on the underlying mechanisms of proliferation inhibition. Although cell cycle analysis showed that IFITM3 knockdown had no effect on cell cycle arrest (Supplemental Fig. 1A, B), but in PC-3 cells it was showed that IFITM3 silencing led to Sub-G1 phase cell accumulation (Fig. 3a). The increase of Sub-G1 phase cells indicates the occurrence of apoptosis, so we further confirmed the number of apoptotic cells by analysis with Annexin-V/7-ADD staining. The percentage of apoptotic cells in PC-3 cells also increased (Fig. 3b), suggesting that proliferate inhibition may cause by increased apoptosis. These results can be considered as strong evidence that IFITM3 plays a key role in PCa cell proliferation.

### Inhibition of IFITM3 reduces invasion and migration of PCa cells

To explore the effects of IFITM3 on tumor cell metastasis and invasion, a transwell migration assay was performed with PC-3 after starvation culture one day, demonstrating the inhibit effect of IFITM3 knockdown on cell invasion and migration. After staining with crystal violet, the number of cells in the Lv-shIFITM3 group in the lower chamber was lower than that in the control group. (Fig. 3c, d): the mean cell count in Lv-shIFITM3 group was 98 compared with 174 for Lv-shCON group. The statistical analysis performed on different views of view randomly selected under the microscope showed a significant decrease (***P < 0.001). In addition, the absorbance at 570 nm in the Lv-shIFITM3 group was also decreased, further suggesting that the cell migration ability was decreased. We also did the above experiment in DU145 cells, however, silencing IFITM3 did not change the invasive ability of DU145 cells (Supplemental Fig. 1C, D), which may be due to the low expression level of IFITM3 in DU145 cells.

Since IFITM3 acts a pivotal role in the malignant proliferation and metastasis of tumor cells, we examined the key molecules of common pathways such as cell cycle, proliferation, and metastasis by using the Western blotting method to explore the relevant mechanism of IFITM3. The MAPK (Mitogen-activated protein kinases), a conserved family of enzymes, including several major components (JNK, P38, and ERK), had previously been found to play important roles in cellular biological processes such as proliferation, apoptosis, stress response, and metabolism^17–21^. We found that in the absence of IFITM3, phosphorylation of P38-AMPK was significantly weakened and the phosphorylation level of CDK1 (CDC2) was also decreased (Fig. 3e), confirming the effect of IFITM3 on tumor cell proliferation. We also found that inhibition of phosphorylation of autophagy-related molecule (mTOR) after IFITM3 knockdown induced apoptosis. The expression of EMT relative protein E-Cadherin and N-Cadherin in cancer cells also changed after IFITM3 was silenced (Fig. 3f), indicating that the metastasis ability of tumor cells was reduced by inhibiting the EMT process.

### IFITM3 regulates tumor cell proliferation and metastasis via TGF-β-MAPK signaling pathway

**Fig. 4:**
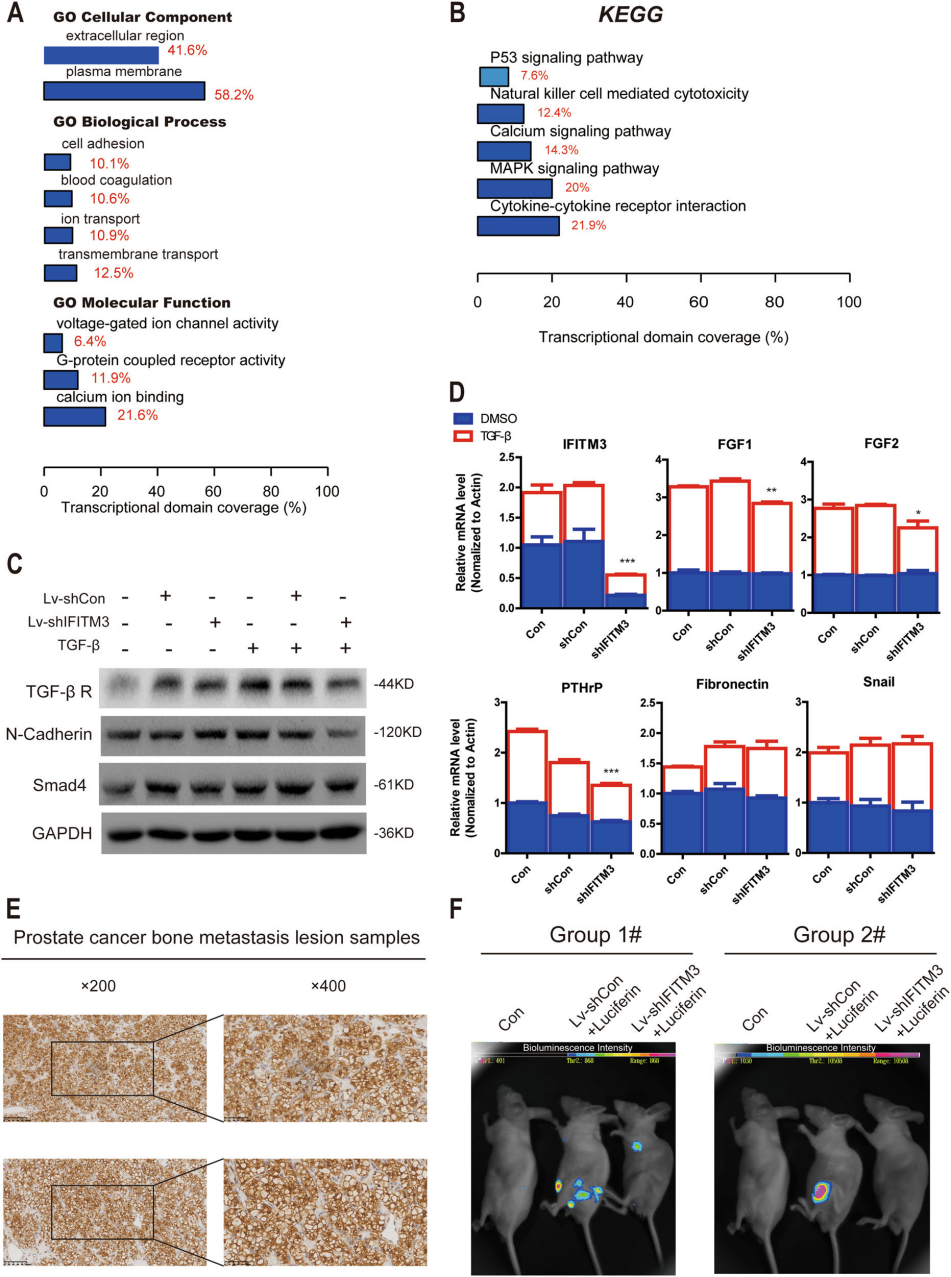
Silencing IFITM3 inhibits TGF-β-induced Smad-MAPK signaling pathway and PCa bone metastases in vivo. **a**, **b** GO and KEGG pathway analysis of microarray results after IFITM3 knockdown. **c** Western blotting was used to analyze protein expression changes of TGF-β Receptor, N-cadherin, and Smad4 with TGF-β (5 ng/mL) incubation for 24 h compared with the normal control. **d** Changes in metastasis-related molecules after knocking down IFITM3 with or without TGF-β (5 ng/mL) added by qPCR analysis. **e** Representative immunohistochemical images are presented about IFITM3 expression in PCa bone metastasis tissues. **f** Metastasis lesions were monitored by bioluminescence intensity in mice after 4 weeks tail vein injection of PC-3 cells

To explore IFITM3-related signaling pathways, PC-3 cells were transduced with shIFITM3 and subjected to microarray analysis. GO analysis (Fig. 4a) revealed that IFITM3 participated in cell adhesion (10.1%) and transmembrane transport (12.5%) mainly by affecting G-protein coupled activity (11.9%) mostly in the extracellular (41.6%) and plasma membrane (58.2%). KEGG pathway analysis suggested that MAPK (20.0%) and P53 (7.6%) pathways were part of the five most significant pathways (Fig. 4b). Based on the above bioinformatics analysis results, we believe that changes in TGF-β signaling pathway and related molecules could simultaneously meet the corresponding biological function and signal pathway changes. Here, we hypothesize that TGF-β is a major altered signaling pathway after IFITM3 silencing: IFITM3 knockdown may participate in tumor cell adhesion and division by affecting TGF-β signaling and regulating P53 and MAPK signaling through TGF-β inactivation, which affected the malignant proliferation and metastasis of PCa cells.

### IFITM3 silencing decreases metastasis in TGF-β activating cells and inhibits PCa bone metastasis in vivo

To further determine the effect of IFITM3 on TGF-β and its downstream pathway proteins, we examined the expression of the TGF-β receptor and its complex protein molecule Smad4 after knockdown of IFITM3 in the presence or absence of exogenous TGF-β. We found that the expression of Smad4 and TGF-βR was downregulated relative to the control group with IFITM3 silencing either in the presence or absence of additional TGF-β stimulation, but this trend was more pronounced in the presence of additional TGF-β stimulation. Meanwhile, the expression level of N-cadherin was also consistent with the above performance, indicating that activation of the TGF-β pathway markedly downregulated the expression of N-cadherin after knockdown of IFITM3 (Fig. 4c). All those results suggest that IFITM3 may regulate the invasion and metastasis of cells in the TGF-β-activated state. To confirm the effect of IFITM3 on the migration of PCa cells under different TGF-β activation conditions, qRT-PCR was used to detect the expression levels of metastasis-associated molecules, such as fibroblast growth factor (FGFs), parathyroid hormone-related peptide (PTHrP), fibronectin, and snail. In the experimental group (DMSO), no significant change in the expression level of these molecules was observed compared with the other two control groups after IFITM3 knockdown. However, when TGF-β was added to activate the corresponding pathway, the expression level of FGF1, FGF2, and PTHrP was decreased significantly after silencing IFITM3 (Fig. 4d). After immunohistochemical staining of the PCa bone metastasis tissue samples, we found that the staining of IFITM3 was strongly positive (Fig. 4e), indicating that IFITM3 may be involved in PCa bone metastasis in vivo. Knowing that tail vein injection of PC-3 cells into immunodeficient mice can cause metastasis formation, we used fluorescence imaging to monitor metastasis development in mice. Four weeks later after the tail vein injection with cells, the imaging result showed the fluorescence intensity and range were significantly reduced in sh-IFITM3 group as compared with shCON group, suggesting that cell migration was significantly inhibited in vivo by IFITM3 knocking down. (Fig. 4f).

### Overexpression of IFITM3 promotes tumor cell migration in PCa

**Fig. 5:**
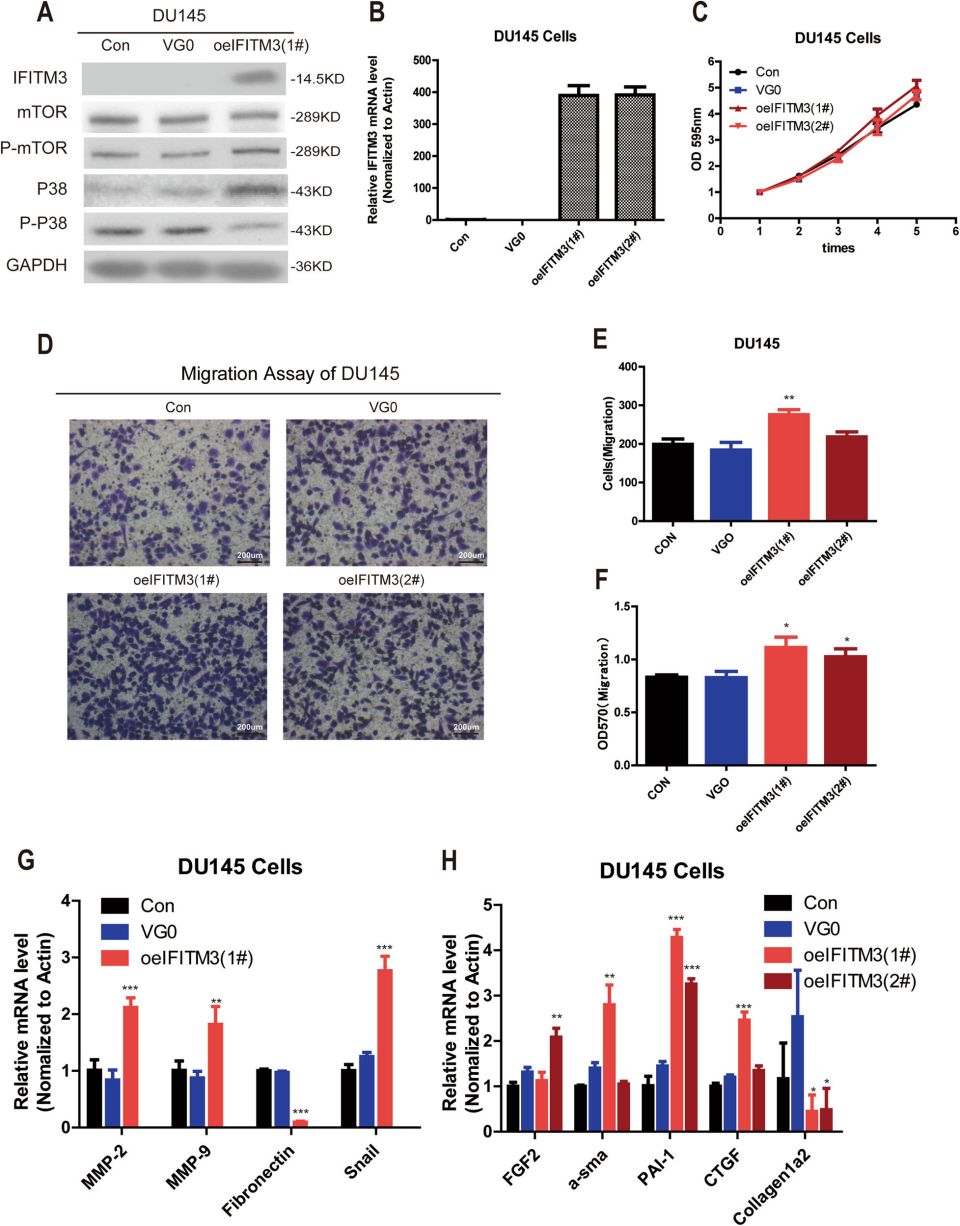
Overexpression of IFITM3 promotes tumor cell metastasis. **a**, **b** The overexpressing efficiency of IFITM3 assessed by qPCR and Western blot, and mTOR/p-mTOR, P38/P-P38 proteins were also analyzed by Western blot in DU145 cells. **c** MTT shows no significant difference in DU145 cell proliferation in all groups. **d** Transwell migration assay demonstrated that the number of cells migrating through the 8 μm diameter pores in both oeIFITM3(#1) and oeIFITM3(#2) groups was increased significantly as compared with the control group. **e**, **f** DU145 cell counts and the absorbance at 570 nm of Lv-oeIFITM3 group were significantly higher than those in the control group. **g** The expression of snail, MMPs, and fibronectin in EMT pathway changed after IFITM3 overexpression. **h** Changes in metastasis-related molecules after overexpressing IFITM3 by qPCR analysis

To demonstrate the relevant function of IFITM3, we performed exogenous overexpression in DU145 cells because of its relatively low basal levels of IFITM3. The overexpression efficiency of IFITM3 gene was detected by Western blotting. The results showed that the expression of IFITM3 protein in Lv-oeIFITM3(#1) group was significantly higher than that in Lv-oeCON(VG0) group in DU145 cells (Fig. 5a). Meanwhile according to the qRT-PCR results, the expression of IFITM3 gene in DU145 cells was increased both in Lv-oeIFITM3(#1) and Lv-oeIFITM3(#2) groups (Fig. 5b). The above results demonstrated that IFITM3 was overexpressed efficiently and could be used in the subsequent experiments. Then, we used MTT method to evaluate cell proliferation (Fig. 5c). Compared with the control group, neither Lv-oeIFITM3 (#1) nor Lv-oeIFITM3(#2) showed a significant difference. Subsequently, the transwell migration assay was performed again to elucidate the effect of the IFITM3 gene on cell migration and invasion. It was showed that an increased number of cells in the Lv-oeIFITM3 group at the bottom of the filter compared to the other two control groups (Fig. 5d). The randomly chosen views observed by light microscopy showed that the mean cell count of DU145 in Lv-oeIFITM3 (#1) group was increased significantly as compared with Lv-shCON group (**P < 0.001) (Fig. 5e). Furthermore, the number of cells confirmed by absorbance at 570 nm in Lv-oeIFITM 3 (#1) and Lv-oeIFITM 3 (#2) increased significantly (*P < 0.05) (Fig. 5f). qRT-PCR revealed that after IFITM3 overexpression, the EMT related pathway genes expression such as MMP-2, MMP-9, fibronectin, and snail was significantly upregulated in PC-3 cells (Fig. 5g). Likewise, the expressions of these protumor metastasis factors also underwent significant changes, further validating that IFITM3 promotes tumor metastasis (Fig. 5h).

### IFITM3 affects PCa bone metastasis by binding to Smad4 via the TGF-β-Smads-MAPK signaling pathway

**Fig. 6:**
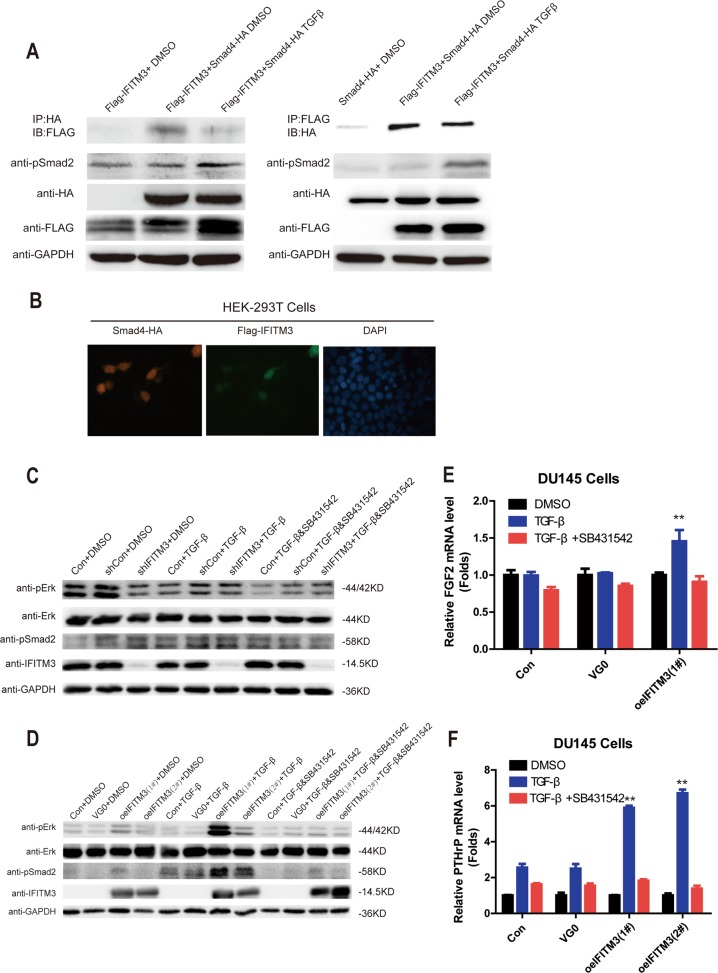
IFITM3 affects PCa bone metastasis through regulation of The TGF-β-induced Smads signaling pathway. **a** Western blot analysis of an anti-HA or anti-FLAG coimmunoprecipitation (IP) experiment performed in cell extracts containing transiently expressed FLAG-IFITM3 and Smad4-HA. **b** The lentivirus was successfully transfected and the result was measured in fluorescence (magnification × 100). **c**, **d** Changes in ERK and Smad2 phosphorylation levels after IFITM3 knockdown or overexpression as shown by Western blot analysis. 10 μM SB431542 significantly reversed the enhancing expression of p-Smad2 and p-ERK induced by TGF-β (5 ng/mL) in IFITM3 overexpressed DU145 cells. The coordination of TGF-β and SB431542 is shown. **e**, **f** SB431542 (10 μM) significantly reversed the mRNA expression of FGF2 and PTHrP induced by TGF-β (5 ng/mL) in oeIFITM3 groups

The Smads family of proteins plays a key role in the transduction of TGF-β signals from cell surface receptors to the nucleus and different Smads mediate signal transduction of different members of the TGF-β family. Co-Smad, including Smad4, is a common mediator of various types of signaling in the TGF-β family. Due to the close relationship between IFITM3 and TGF-β signaling pathway, we hypothesized that there may be an interaction between IFITM3 and one of the Co-Smads. To identify the molecular interaction between Smad4 and IFITM3, Western blot analysis of anti-FLAG and HA co-immunoprecipitation (IP) experiments was performed in HEK-293T cell extracts containing transiently expressed FLAG-IFITM3 and HA-Smad4 (Fig. 6a). The Co-IP assay suggested that IFITM3 could directly bind to Smad4, and the expression of Smad4 downstream molecule p-Smad2 known as an indicator of Smads signaling pathway activation was increased simultaneously in the presence of additional TGF-β stimulation, suggesting that IFITM3 and Samd4 played a synergetic physiological function. Cultured cells were transfected with a plasmid containing expression for Smad4-HA (cherry fluorescence) or expression plasmids encoding FLAG-IFITM3 (green fluorescence). DNA was visualized with DAPI (Blue fluorescent) by immunostaining (Fig. 6b). According to the fluorescence intensity, it was found that plasmid transfection was successful. Both exogenous IFITM3 and Smad4 were expressed continuously in transfected cells. To further clarify the effect of IFITM3 on TGF-β-MAPK-Smads pathway, we detected the key pathway molecules (ERK, Smad2) by IFTM3 knockdown and overexpression, respectively. The results suggest that phosphorylation of ERK and Smad2 was inhibited in the IFITM3-silenced state, but the inhibition was not significantly different with or without TGF-β (Fig. 6c). In the case of exogenous IFITM3 overexpression, although the phosphorylation activation level of ERK and Smad2 was increased, the level of phosphorylation of ERK and Smad2 increased more significantly compared with the DMSO-added control group, and this trend could be basically reduced to that of the control group after addition of the specific TGF-β receptor inhibitor SB431542 (Fig. 6d). Finally, we detected the expression of FGF2 and PTHrP by qRT-PCR, knowing that they are representative molecules of PCa bone metastasis. It was found that the expression of FGF2 and PTHrP mRNA was significantly increased after IFITM3 overexpression compared with the two control groups, suggesting that overexpression IFITM3 could promote PCa bone metastasis (Fig. 6e, f).

## Discussion

**Fig. 7:**
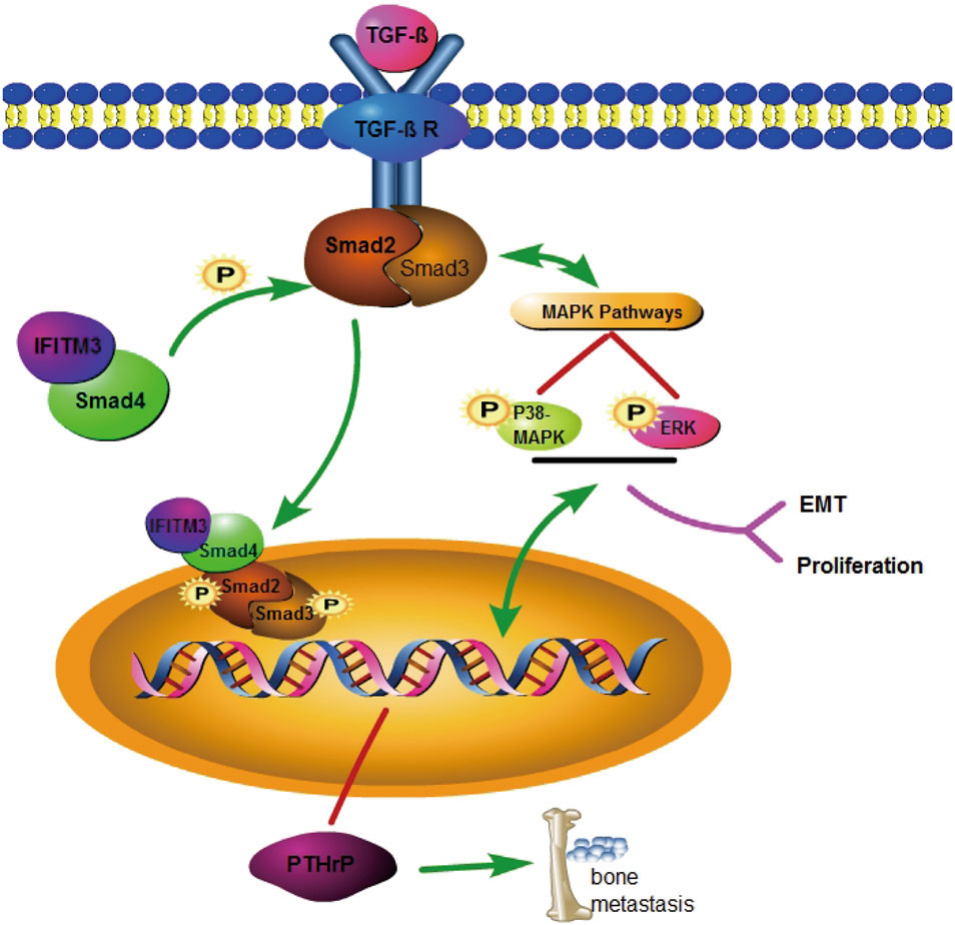
The promotor role of IFITM3 in PCa cell proliferation and metastasis. Overexpression of IFITM3 combined with Smad4 promoted Smad2/3 phosphorylation, thereby affecting transcriptional regulation to induce PTHrP-driven bone metastasis. In addition, the TGF-β-Smads pathway had crosstalk with MAPK pathway, thus promoting malignant proliferation of tumor cells by activating the p38-MAPK and ERK pathways, and simultaneously regulating gene transcription level and EMT to facilitate tumor cell metastasis

Many previous studies have demonstrated that IFITM3 is a tumor-promoting gene overexpressing in many malignancies, including gliomas, colorectal cancer, and breast cancers^12,15,16^. However, the clinical value and downstream molecular mechanisms of IFITM3 in PCa remain elusive so far. To investigate the IFITM3 expression in PCa tissues, we performed qRT-PCR analysis, immunohistochemical staining, and ONCOMINE data mining and found abnormally high expression levels of IFITM3. More importantly, the expression of IFITM3 was positively correlated with Gleason score and T stage. Subsequently, by using a lentiviral vector tool encoding siRNA against IFITM3, we found that IFITM3 knockdown inhibits cell proliferation and induces apoptosis. By examining the key molecules of common pathways such as cell cycle, proliferation using the Western blotting method, we found that P38-MAPK activation was significantly weakened and the expression level of cell cycle-related proteins CDK1 and CDC2 was decreased, suggesting that IFITM3 may affect the proliferation of PCa cells through regulating cell cycle mediated by MAPK pathway. Meanwhile, we found that the E-Cadherin and N-Cadherin changed accordingly after silence IFITM3, knowing that E-cadherin loss in epithelial cell adhesion is a characteristic feature of EMT, which allows to enhance cells movement and aggressiveness by disrupting cell-cell contacts^22^. EMT is one of the key processes in PCa invasion and metastasis, which allows epithelial cells to break through the basement membrane and further gain the ability to metastasize to the distal site^23^. Meanwhile, MAPK is also known to be participated in the regulation of the EMT process^24,25^, indicating that the regulatory function of IFITM3 on proliferation and metastasis of PCa cells may associate with MAPK pathway (Fig. 7).

Subsequently, we performed microarray analysis and found that IFITM3 participated in regulating tumor cell proliferation and metastasis via TGF-β-MAPK signaling pathway, which is consistent with most results obtained from KEGG and Gene Ontology analysis. More significantly, we found that knockdown of IFITM3 inhibited the metastasis-associated molecules (including EMT and bone metastasis). Especially in the presence of exogenous TGF-β stimulation, this inhibitory effect was more pronounced. Meanwhile, we used the lentiviral vector to infect DU145 cells for exogenous overexpression of IFITM3, and found that IFITM3 overexpression promoted cell migration, which was accompanied with a significant increase in metastasis-related molecules. Knowing that enrichment of TGF-β is a characteristic microenvironment before PCa metastasis, we focused on the function of IFITM3 in TGF-β-stimulated regulation of metastases including bone metastasis and EMT-related processes through the regulation of the TGF-β-MAPK pathway.

Tumor metastasis is the most important biological characteristic of malignant tumors, and is also the main reason for treatment failure and the patient death. Recent studies have revealed that TGF-β act a pivotal part in the development of bone metastasis in BCa and PCa. TGF-β family members signal through membrane-bound, heteromeric serine-threonine kinase receptor complexes, resulting in phosphorylation of the Smad family proteins through the activation of TGF-β ligands. The phosphorylated Smad family proteins accumulated in the nucleus and acted as transcription factors to regulate the expression of the target genes, either directly on the DNA of SMAD-specific cis-elements or through physical interactions with other transcription factors acting on their cognate DNA recognition sites^26^.

The abundant TGF-β in the bone matrix is one of the important growth factors affecting bone metastasis. Transfer of cells to bone tissue releases a prometastatic factor that activates osteoclast differentiation. Once this process is activated, osteoclasts degrade the bone matrix and release large amounts of TGF-β. Histological analysis showed that 75% of human bone metastases pathologically found phosphorylated Smad2 in the nucleus of the metastatic cells, indicating that the TGF-β signaling pathway is activated and functions^27^. Knockdown of Smad4, inhibition of Smad7 expression, and expression of a dominant-negative TGFBR2 can all block the TGF-β signaling pathway and significantly reduce bone metastasis in breast and melanoma models, further giving evidence of that the TGF-β signaling pathway participate in bone metastasis^28^. TGF-β can also promote the expression of bone resorption factors such as PTHrP and receptor activator of nuclear factor kappa B ligand(RANKL) via Smad and MAPK signaling pathways. PTHrP induced by TGF-β can reduce the expression of osteoprotegerin and promote bone matrix dissolution and bone metastasis by increasing the expression of RANKL in osteoblasts and stromal cells^29^.

Smads-dependent signaling is a classical pathway mediated by TGF-β. The TGF-β superfamily ligands first bind to a type II receptor then recruit and phosphorylate the type I receptor. Subsequently, the type I receptor regulates phosphorylation of the Smad2/3 complex that binds to Smad4.P-Smads/Smad4 complexes as a transcription factor which accumulate in the nucleus and participates in the regulation of EMT and bone metastasis target gene expression^30,31^. Under the resting state, Smad2 is unphosphorylated and remains in the cytoplasm. After TGF-β stimulation, Smad2 is phosphorylated and dimerized with Smad3, allowing it to translocate to the nucleus^32^. The result of Co-IP method confirmed that IFITM3 could bind to Smad4 directly and affect the phosphorylation of Smad2, which reveals a new way of modulating the TGF-β pathway by IFITM3. Then, we used a specific inhibitor of TβRI kinases, SB431542^33^ to inhibit TGF-β induced phosphorylation of Smad2. When inhibiting the function of TβRI kinases by SB431542, it could weaken the phosphorylation level of ERK and Smad2 and make it return to the level of the control group after IFITM3 overexpression, while overexpression of IFITM3 could significantly enhance the phosphorylation activation level of ERK and Smad2 induced by TGF-β. Those results revealed that IFITM3 may be the important regulating point of TGF-β-Smads-MAPK signaling pathway in promoting EMT and bone metastasis induced by TGF-β in PCa cells.

To sum up, the present study revealed that IFITM3 was an oncogene in PCa cells and could promote EMT and bone metastasis mediated by TGF-β. Furthermore, experiments in vitro and in vivo have demonstrated that abnormal IFITM3 expression enhanced the invasion ability of PCa cells. IFITM3 interacts with Smad4 and affects the phosphorylation activation of Samd2, which is why IFITM3 functioned more strongly under TGF-β stimulation. The mechanism of IFITM3 on the regulation of the TGF-β-Smads-MAPK pathway may contribute to a deeper understanding of PCa progression and provide new ideas for the treatment strategies for PCa patients with bone metastasis.

## Supplementary information

Supplemental Figure 1

Supplementary figure legends
