# Supplementary figures and images for: IFITM3 promotes bone metastasis of prostate cancer cells by mediating activation of the TGF-β signaling pathway
Source: Cell Death Dis. 2019 Jul 4;10(7):517. doi: 10.1038/s41419-019-1750-7 (PMC6609682; doi:10.1038/s41419-019-1750-7)

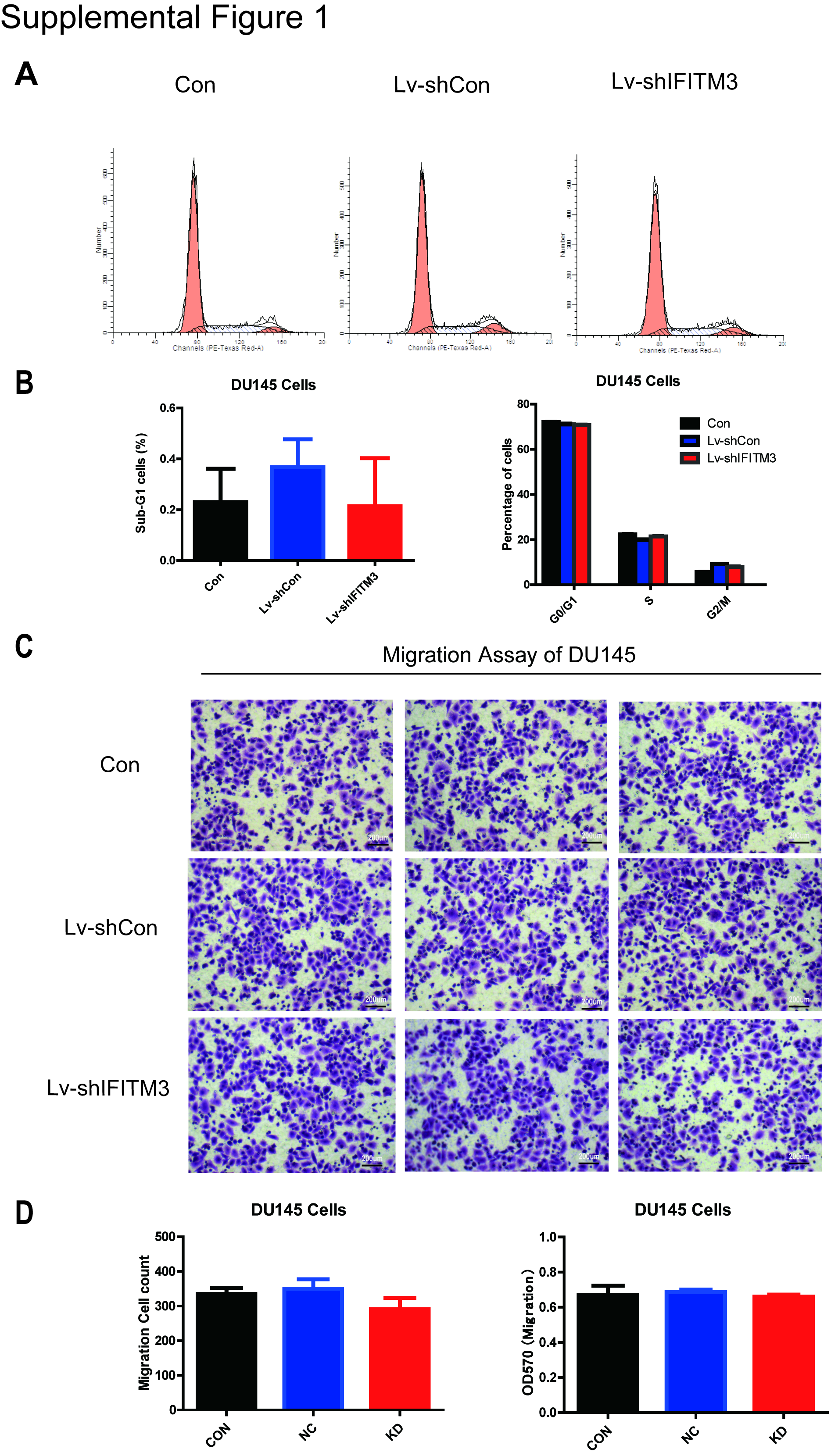

Supplement: Supplementary file 1 — Supplemental Figure 1 [file 41419_2019_1750_MOESM1_ESM.tif]
