# Supplementary material for: IFITM3 promotes bone metastasis of prostate cancer cells by mediating activation of the TGF-β signaling pathway
Source: Cell Death Dis. 2019 Jul 4;10(7):517. doi: 10.1038/s41419-019-1750-7 (PMC6609682; doi:10.1038/s41419-019-1750-7)
Supplement: Supplementary file 2 — Supplementary figure legends [file 41419_2019_1750_MOESM2_ESM.docx]

**Supplemental Figure 1. The disruption of IFITM3 expression had no significant effect on the proliferation and invasion in DU145 cells.** (A) Flow cytometry analysis showing cell cycle distributions of DU145 cells between Con, Lv-shCon and Lv-shIFITM3 groups. (B) Percentages of DU145 cells infected by Lv-shCon or Lv-shIFITM3 in G0/G1, S, G2/M and Sub-G1 phases were analyzed statistically. (C) Transwell migration assay showed that the number of cells migrating through the 8um diameter pores in each groups. (D) Cell counts has no significantly difference in each groups. The absorbance of Con, Lv-shCon and Lv-shIFITM3 groups at 570nm objective indicate the number of metastatic cells.
